# Cost-effectiveness of laparoscopic cholecystectomy in high-altitude areas

**DOI:** 10.1097/MD.0000000000045644

**Published:** 2025-11-07

**Authors:** Xiaofeng Jing, Ying Ma, Defu Li, Tiecheng Zhang, Haiqi Xiang, Fan Xu, Yonghong Xia

**Affiliations:** a Department of Public Health, Chengdu Medical College, Chengdu, Sichuan, China; b Department of Hospital Infection Control, Jintang First People’s Hospital, Chengdu, Sichuan, China; c Department of Hepatobiliary Surgery, Qinghai Traffic Hospital, Xining, Qinghai, China; d Sichuan Provincial Key Laboratory of Philosophy and Social Sciences for Intelligent Medical Care and Elderly Health Management, Chengdu Medical College, Chengdu, Sichuan, China; e Irradiation Preservation and Effect Key Laboratory of Sichuan Province, Chengdu, Sichuan, China.

**Keywords:** cost-effectiveness, gallstone, high-altitude area, laparoscopic cholecystectomy

## Abstract

**Background:**

At present, laparoscopic cholecystectomy (LC) is the primary treatment for gallstone. Although the application of LC has been widespread in developed countries, the cost-effectiveness of LC in high-altitude, low-resource regions remains unclear.

**Methods:**

We aimed to determine the cost-effectiveness of LC in the Qinghai Province and to provide a reference for other high-altitude area. We included 124 patients treated from 2018 to 2022 in Qinghai Traffic Hospital. We collected their demographic and clinical information and used the EuroQol Five Dimensions questionnaire to assess their quality of life. We performed cost-effectiveness analysis to evaluate LC and open cholecystectomy (OC). We also performed sensitivity and threshold analyses to determine the robustness of the results.

**Results:**

The OC and LC group demonstrated no significant differences in demographic characteristics. However, the hospital costs, the length of stay and quality-adjusted life years were significantly different between 2 groups (*P* < .05). The average cost was $1293 for LC and $2480 for OC. When the willingness to pay value is $2328 per quality-adjusted life year, the probability is >0.9, while when the willingness to pay value is $2793 per length of stay, the probability is >0.8.

**Conclusion:**

LC is less expensive and more effective than OC from the patient and society perspectives. Although there are some obstacles, it is feasible to promote LC in high-altitude, low-resource areas.

## 1. Introduction

Open cholecystectomy (OC) used to be the gold standard treatment for gallstone until the 1980s: the introduction of laparoscopic cholecystectomy (LC)^[1]^ has relegated OC to an alternative operation. LC is now the primary treatment for complicated gallstone disease in high-income countries. It is associated with a shorter length of stay (LOS), a smaller incision, a more rapid recovery, lower mortality and significantly reduced hospital medical costs compared with OC.^[2]^ LC has some high initial costs for the equipment, infrastructure and disposable medical materials, so cost-effectiveness has been the major obstacle to widespread acceptance of LC.^[3]^ Nevertheless, the benefit of LC has still led high-altitude areas to promote the use of LC.

Researchers have demonstrated that it is feasible to promote LC in high-altitude, low-resource areas. It was safe to perform LC in Afghanistan and it had comparable outcomes in terms of complications and conversion rates to OC as other countries.^[4]^ A study in Chile also demonstrated that LC reduced the LOS in patients with mild gallstone pancreatitis.^[5]^ Researchers have also investigated the application of laparoscopic surgery for other disease in Qinghai Province. They found that laparoscopic surgery is superior to traditional open surgery in terms of the LOS, the recovery time, complications, and the risk of incision infection.^[6]^ While it is possible to promote laparoscopic surgery in developing high-altitude areas, there has been a limited focus on the cost-effectiveness of this approach.

Overall, access to essential health services has improved from 2000 to 2017, but service coverage in low- and middle-income countries has remained much lower than in high-income countries. In 2017, only one-third of the world’s population had access to basic sanitation, and the inability to pay for health care remained a major challenge.^[7]^ Endoscopy through a natural orifice has become the latest technique in the field of surgery, and many Chinese surgeons have been involved in several surgeries that use this approach.^[8]^

Due to the long, cold winter, people who live in Qinghai-Tibet Plateau consume a high-energy diet, which leads to a high incidence of gallstone.^[9]^ Given this high incidence, a more cost-effective approach is needed to reduce the medical burden of patients. The high initial capital costs, infrastructure deficits and lack of the health professionals are barriers to the widespread use of LC in Qinghai-Tibet Plateau. To ensure resources are used efficiently in this region, we determined the cost-effectiveness differences between OC and LC and patient selection preference at Qinghai Traffic Hospital in the Qinghai-Tibetan Plateau. Our findings should support provide reference for the use of LC in other high-altitude, low-resource areas.

## 2. Methods

### 2.1. Design

We conducted this study considering patients treated at Qinghai Traffic Hospital, Xining, Qinghai. This hospital was a tertiary Grade A nonprofit medical institution, also designated as a national emergency rescue center network hospital by the National Health Commission. It provided health care services to whole provincial patients and has a capacity of approximately 700 beds.

### 2.2. Ethic statement

This research conforms to the ethical guidelines of the Declaration of Helsinki. The study was approved by the Institutional Review Board and by the Ethics Committee of Qinghai Provincial Traffic Hospital (approval no. 20221201007). All participants provided written informed consent.

### 2.3. Participants

We screened patients aged 20 to 84 years with a clinical diagnosis of cholelithiasis and who had undergone OC or LC between May 2018 and August 2022 for eligibility. The inclusion criteria were: age ≥ 18 years old, gallstone diagnosed using transabdominal ultrasonography (no history of abdominal surgery). The exclusion criteria were: pregnancy, suspected or confirmed malignancy, incomplete magnetic resonanced cholangio-pancreatography and contraindication for laparoscopy. We selected 3886 patients who were admitted to the general surgery department. After screening the patients based on baseline information, the simple random method was used with random seed 202,212 to randomly selected 124 patients. There were 62 eligible patients in the OC group and 62 eligible patients in the LC group.

### 2.4. Outcome

The primary outcome was incremental dollars per hospital day averted, which indicates the additional cost incurred to avoid 1 day of hospital stay by using LC instead of OC. We compared cost-effectiveness between the OC and LC groups and against the Qinghai Province gross domestic product per capita. The second outcome for the cost-effectiveness analysis was quality-adjusted life years (QALYs). QALY refers to life years weighted by the quality of life (QOL) value, a QOL is a generic measure of disease burden: 0 means death, while 1 indicates perfect health. For the cost-utility analysis, we determined QOL based on the converted value of the EuroQol Five Dimensions questionnaire (Chinese version).^[10,11]^

### 2.5. Cost

We measured costs in U.S. dollars ($). We collected the following costs primarily from electronic medical records: surgery, computed tomography, pathological examination, ultrasound, accommodation, electrophysiology, radiation, nursing, laboratory inspection, anesthesia, heating, blood transfusion, oxygen delivery, examination, traditional Chinese medicine, magnetic resonance imaging, endoscopy, medical material, medicine, and consultation. The average total costs were $1293 for LC and $2480 for OC. All costs were retrospectively audited by a blinded assessor through the hospital’s electronic medical record system after the patient was discharged from the hospital.

### 2.6. Effectiveness

For the cost-effectiveness analysis, we calculated the incremental cost and effectiveness of the LC group compared with the OC group. Incremental costs are the mean difference between both groups in total costs over whole progress of hospitalization. Incremental effectiveness is the mean difference in the QALYs/LOS over hospitalization. For the CUA, we calculated the incremental cost utility as the difference in the total costs divided by the difference in QALYs. The incremental cost-effectiveness ratios are stated in terms of costs per unit of LOS and costs per QALY.^[12]^

### 2.7. Analysis

We performed all analyses according to the intention-to-treat principle. We recorded and stored the data in Excel files and analyzed it using Stata 15.0 (StataCorp LLC). We compared demographic information between 2 groups by using the Chi-square test. We compared differences in surgical approach between the groups by using 2-tailed Student *t* test. We considered *P* < .05 to indicate a statistically significant difference.

We used bootstrapping to deal with the great heterogeneity in the cholecystectomy-related hospitalization costs, outcomes, and effectiveness. This method empirically estimate the sampling distribution by resampling from the original data with replacement, without assuming a specific distributional form like a normal distribution based on mean and variance. Specifically, we ran the economic and clinical outcomes of the original trial through a mathematical model that uses the variance of the original data to simulate hypothetical outcomes after 100s or 1000s of runs of the same trial. The cost-effectiveness ratios of these simulations better represent the wide range of cost-effectiveness outcomes that are possible in a population, rather than just those sampled in a trial.

Using Monte-Carlo simulation, we conducted a probabilistic sensitivity analysis that ran 1000 iterations of the model to generate a cost-effectiveness scatterplot and a cost-acceptability curve with a willingness to pay threshold of $2590 (calculated by per capita gross domestic product of Qinghai Province, 2023).

## 3. Results

We included 124 patients in the analysis and were significantly different between the OC and LC groups. There was no significance between 2 groups in demographic characteristics such as sex, age, insurance type, ethnicity, and discharge outcome (*P* > .05). LC was less expensive and more effective than OC. Patients who underwent LC had lower total medical costs (*P* = .000). OC resulted in an average QALY gain of 0.73 per patient, whereas LC provided an average QALY gain of 0.82 per patient. The average LOS of patients who underwent OC was longer than for patients who underwent LC (Table 1).

**Table 1:** Demographic characteristics of patients who received open cholecystectomy (OC) and laparoscopic cholecystectomy (LC).

	All	OC	LC	*P*
*Sex*				.284
Male	59	32	27	
Female	65	29	36	
*Ethnicity*				.402
Han	77	38	39	
Tibetan	37	20	17	
Hui	10	3	7	
*Insurance*				.468
Urban workers	50	23	27	
Urban residents	25	11	14	
Rural	44	23	21	
Self-paying	5	4	1	
*Discharge outcome*				
Cure	123	61	62	.323
Death	1	0	1	
*Age in years (mean ± SD*)	57.98 ± 1.60	54.03 ± 1.74	54.03 ± 1.64	.088
*Cost in U.S. $ (mean ± SD*)	1923.50 ± 115.77	2553.51 ± 194.73	1331.20 ± 70.34	.000*
*LOS in days (mean ± SD*)	9.53 ± 0.71	13.01 ± 1.28	6.16 ± 0.27	.000*
*QALY (mean ± SD*)	0.78 ± 0.01	0.73 ± 0.02	0.82 ± 0.02	.000*

LOS = length of stay, QALY = quality-adjusted life year, SD = standard deviation.

* *P* < .05.

### 3.1. Sensitivity analysis

**Figure 1.**
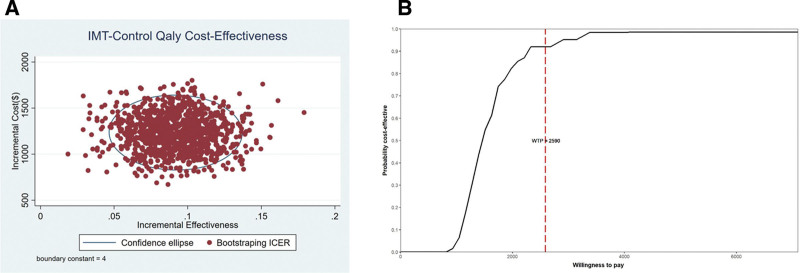
(A) Incremental cost-effectiveness plane of quality-adjusted life years (QALYs). The circle represents the 95% confidence interval. (B) Cost-effectiveness acceptability curve of QALYs.

**Figure 2.**
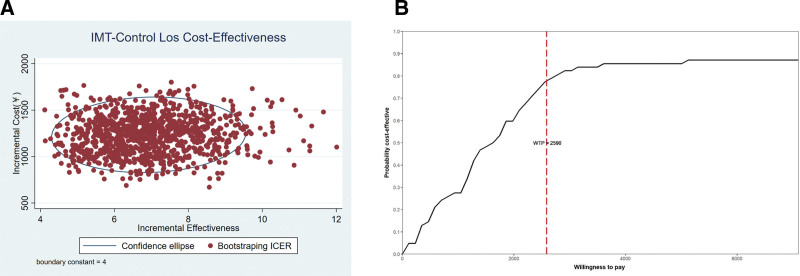
(A) Incremental cost-effectiveness plane of the length of stay (LOS). The circle represents the 95% confidence interval. (B) Cost-effectiveness acceptability curve of LOS.

The 1000 bootstrap iterations are displayed in the cost-effectiveness plane, illustrating the uncertainty regarding the difference in costs and QALYs (Fig. 1) costs and LOS (Fig. 2) between the OC and LC groups. The cost-effectiveness plane shows the difference in costs and QALYs gained for the interventions for each of the 1000 model runs (Fig. 1A). The points are concentrated in one quadrant of the plane, indicating that LC is more effective and costs less. The cost-effectiveness acceptability curve shows that LC is cost-effective across a range of willingness-to-pay values for QALYs. At a threshold of $2590 per QALY, LC is cost-effective for over 90% of simulations (Fig. 1B). Besides, the cost-effectiveness plane in Figure 2A shows the difference in cost and LOS between LC and OC. Similarly to Figure 1A, the simulated points are concentrated in one quadrant of the cost-effectiveness plane, indicating that LC has a faster recovery rate and is much less expensive than OC. The cost-effectiveness acceptability curve in Figure 2B shows the LC is cost-effective across a range of willingness-to-pay values for the LOS. At a threshold of $2590 per LOS, LC is cost-effective for >75% of simulations.

## 4. Discussion

The main purpose of this study was to determine whether LC is better than OC in terms of costs, effects and utility from the clinical and societal perspectives. The average cost was $1293 for LC and $2480 for OC. We found that LC may be a more effective and affordable approach. When the intention to pay is between $100 and $900 per QALY, the probability changes from 0 to 0.016. However, when the willingness to pay value is $2328 per QALY, the probability is >0.9. Besides, when the intention to pay is between $0 and $100 per LOS, the probability changes from 0 to 0.048, while when the willingness to pay value is $2793 per LOS, the probability is >0.8. These data indicate LC is more effective when considering recovery and quality of life.

As summarized in Table 2, the medical burden associated with OC extends beyond direct hospital costs. LC offers a clear advantage in reducing costs across multiple dimensions, including direct hospitalization expenses,^[13–15]^ indirect costs incurred by patients and families,^[16–18]^ the strain on the healthcare system and societal burden.^[19,21–23]^ These cost reductions are especially significant in the high-altitude, resource-limited area. The lower incremental cost-effectiveness ratios for OC compared with LC may be correlated with several factors. In one study, LC was associated with a significant reduction in infections, a phenomenon related to the smaller incision and shorter operative time for LC.^[24]^ Similarly to Coccolini et al,^[22]^ our analysis of hospital stays showed that patients who accepted LC had a shorter LOS than OC, a mean difference of 3.48 days. The shorter LOS demonstrates the advantages of LC in reducing pain and improving the recovery rate. LC had lower total medical expenses than OC. Although LC has higher disposable material and equipment costs than OC, LC is less expensive in terms of other costs like medication. In our study, the operative and postoperative care costs of LC were lower than for OC. As the use of LC has increased, the equipment costs for this procedure have decreased.^[13]^ The scar of LC is smaller and more aesthetically acceptable. A study found high patient satisfaction of LC especially among unmarried and <40-year-old women.^[25]^ In our patient cohort, more women chose the LC approach, perhaps reflecting that women preferred the postoperative aesthetic outcome.

**Table 2:** The comparison of medical burden between LC and OC.

Burden category	Component	Comparison: LC vs OC	Evidence/implication
Direct medical costs	Hospitalization costs	LC lower than OC ($1293 vs $2480)	Reduced financial burden on patients and insurance funds.^[13]^
	Surgical team	OC require more experienced surgical team	Male preference for OC may link to fibrosis, abnormalities, and team experience.^[14]^
	Surgical/disposable costs	LC was relatively higher at the initial setup	The cost of the surgical equipment for LC was relatively high at the beginning and decreased as the number of uses increased.^[15]^
Indirect patient/family costs	Lost wages (patient)	Shorter LOS enables faster return to work (6.03 d vs 13.16 d)	Significant factor in low-resource settings where daily wages are crucial.^[16]^
	Caregiver costs	Less human and time costs for care	Reduces burden on family support systems.^[17]^
	Patients life quality	LC offers better postoperative recovery and shorter follow-up (0.82 vs 0.73).	The patients with a symptomatic gallstone disease report they feel better after LC.^[18]^
Healthcare system burden	Bed occupancy	LC is shorter in LOS	Improves hospital efficiency and resource availability in constrained settings.^[19]^
	Readmission rate	Readmissions rates following LC are relatively low	Besides surgery, patient insurance status also impacts readmission rates.^[20]^
	Complication management	LC have potentially fewer complications	Postoperative wound infection, pneumonia rates and operative mortality rate were reduced by LC.^[21]^
Societal burden	Productivity loss	Faster recovery to full function with LC	Contributes to overall economic well-being in the region.^[22]^

LC = laparoscopic cholecystectomy, LOS = length of stay, OC = open cholecystectomy.

Previous studies have reported mixed results regarding whether implementing LC in developing regions is cost-effective.^[26,27]^ There has been support for LC, while other researchers believe that LC is only cost-effective for high-income countries until new cost-reduction strategies are introduced.^[28]^ We found that LC was less expensive than OC in Qinghai Province, but it is worth noting that there are still many obstacles to the promotion of LC in developing, high-altitude area, including a lack of training, functioning equipment and qualified surgeons.^[29,30]^ In developing areas, the local residents may have relatively poor health awareness and hygiene conditions and many patients may not seek medical treatment until the disease is serious. Referral may not always be timely, resulting in a higher proportion of serious cases.^[31]^ In these cases, doctors usually have to perform OC to ensure the safety of the patients.^[32]^ Nevertheless, developing countries such as India have proved LC is a safe, feasible and has potential benefits for health care delivery.^[33]^ The possibility of application of LC has also be confirmed in Mongolia.^[34]^ These success can help to popularize LC.

Furthermore, ablation as a minimally invasive treatment, is often considered an alternative to laparoscopic surgery. In settings where advanced interventional radiology is limited, a well-established laparoscopic ablation program can offer a valuable alternative for managing selected liver tumor.^[35]^ Although the efficiency of radiofrequency ablation for small hepatocellular carcinoma is slightly below laparoscopic surgery, it offers less incisive, lower medical cost and faster recovery rate.^[36]^ Additionally, ablative techniques are increasingly discussed as a potential alternative therapeutic approach to laparoscopic surgery for alleviating pain in endometriosis. Recent studies demonstrated that ablative surgery is equally effective as laparoscopic surgery in relieving long-term pain in patients, while also offering more personalized treatment options.^[37]^ Therefore, future research can explore the popularization and application of radiofrequency ablation in low-resource settings.

Our results allow us to address several outstanding questions. First, is the value of LC good for the health care sector and/or society? Our results suggest that LC is of intermediate-to-high value for gallstone at 2023 prices. Second, is LC affordable to the health care sector/society? After the reform of China’s medical system and the control of indicators, the various costs of LC have been reduced greatly, especially the costs of medicine and surgery, which has reduced the financial burden of medical care in China.^[20]^ We also found that LC cost less than OC. Third, can patients afford LC? In our study, 42.85% patients who chose LC were covered by urban worker insurance, which reimburses about 70% of the medical expenses and thus greatly reduces the medical burden of patients. Under China’s hierarchical diagnosis and treatment system, hospitals of different levels adopt different reimbursement rates for hospitalized diseases: the higher hospital level, the lower the reimbursement rate, and vice versa.^[38]^ In short, if patients can seek medical treatment in a timely manner and adopt LC after the onset of gallstone, it can improve their quality of life and reduce their medical burden.

### 4.1. Limitation

There are costs outside of measurable hospital charges that can be difficult to capture but that affect overall patient outcomes, including time to recovery, return to work and adjuvant chemotherapy. Some studies have shown improved recovery and quality of life after LC,^[30]^ whereas others have demonstrated a shorter time to receipt of adjuvant therapy.^[1]^ These outcomes should undoubtedly be considered a potential cost of each procedure and should be accounted for in surgical decision-making.

## 5. Conclusion

LC is cost-effective from the patient and society perspectives. Although there are some obstacles, it is still feasible to promote LC in high-altitude, low-resource areas.

## Author contributions

**Conceptualization:** Xiaofeng Jing, Ying Ma, Defu Li, Fan Xu, Yonghong Xia.

**Data curation:** Xiaofeng Jing, Ying Ma, Defu Li, Yonghong Xia.

**Formal analysis:** Xiaofeng Jing, Tiecheng Zhang, Haiqi Xiang.

**Funding acquisition:** Yonghong Xia.

**Investigation:** Ying Ma, Defu Li, Tiecheng Zhang, Haiqi Xiang, Fan Xu, Yonghong Xia.

**Methodology:** Xiaofeng Jing, Ying Ma, Defu Li, Tiecheng Zhang, Haiqi Xiang, Fan Xu, Yonghong Xia.

**Supervision:** Ying Ma, Defu Li.

**Validation:** Ying Ma, Defu Li.

**Writing – original draft:** Xiaofeng Jing.

**Writing – review & editing:** Fan Xu, Yonghong Xia.
